# Hydrophobic–Hydrophilic Properties and Characterization of PIM-1 Films Treated by Elemental Fluorine in Liquid Perfluorodecalin

**DOI:** 10.3390/polym14235152

**Published:** 2022-11-26

**Authors:** Nikolay A. Belov, Aleksandr Yu. Alentiev, Dmitrii S. Pashkevich, Fedor A. Voroshilov, Edgar S. Dvilis, Roman Yu. Nikiforov, Sergey V. Chirkov, Daria A. Syrtsova, Julia V. Kostina, Igor I. Ponomarev, Igor P. Asanov, Yulia G. Bogdanova

**Affiliations:** 1 Engineering Center, Tomsk Polytechnic University, 30, Lenin Avenue, 634050 Tomsk, Russia; 2 A.V. Topchiev Institute of Petrochemical Synthesis, Russian Academy of Sciences, 29, Leninskii Prospect, 119991 Moscow, Russia; 3 Institute of Applied Mathematics and Mechanics, Peter the Great St. Petersburg Polytechnic University, 29, Polytechnicheskaya St., 195251 St. Petersburg, Russia; 4 A.N. Nesmeyanov Institute of Organoelement Compounds, Russian Academy of Sciences, 28, Vavilova St., 119991 Moscow, Russia; 5 A.V. Nikolaev Institute of Inorganic Chemistry of Siberian Branch, Russian Academy of Sciences, 3, Academician Lavrentiev St., 630090 Novosibirsk, Russia; 6 Chemical Department, M.V. Lomonosov Moscow State University, GSP-1, Leninskie Gory, 119991 Moscow, Russia

**Keywords:** direct surface liquid-phase fluorination, PIM-1, SEM, XEDS, XPS, ATR-IR, contact angle, surface energy

## Abstract

A direct fluorination technique was applied for the surface treatment of PIM-1 films in a liquid phase (perfluorodecalin). The fluorinated samples were analyzed by various instrumental techniques. ATR-IR spectroscopy showed that the fluorination predominantly takes place in methylene- and methyl-groups. Cyano-groups, aromatic hydrogens and the aromatic structure of the PIM-1 repeat unit were shown to be relatively stable at the fluorination conditions. XPS confirmed that the concentration of fluorine, as well as oxygen, in the near surface layer (~1 nm) increases with fluorination time. C1s and O1s surface spectra of the fluorinated PIM-1 samples indicated an appearance of newly-formed C-F and C-O functional groups. Scanning electron microscopy and X-ray energy-dispersive spectroscopy of the fluorinated PIM-1 samples showed an increase of the fluorine concentration at the surface (~0.1–1 μm) with the treatment duration. Analysis of the slices of the PIM-1 films demonstrated a decline of the fluorine content within several microns of the film depth. The decline increased with the fluorination time. A model of fluorine concentration dependence on the film depth and treatment duration was suggested. A change in the specific free surface energy as a result of PIM-1 fluorination was revealed. The fluorination time was shown to affect the surface energy (γ_SV_), providing its shift from a low value (25 mJ∙m^−2^), corresponding to tetrafluoroethylene, up to a relatively high value, corresponding to a hydrophilic surface.

## 1. Introduction

A process of direct fluorination of polymeric materials has been used to fine tune their surface properties and related functional characteristics [1,2]. It allows for the obtainment of modified fluorine-containing surface layers with an adjustable thickness from 0.01 to 10 microns and chemical composition similar to fluorinated and perfluorinated polymers, while the material bulk properties remain unchanged. A similar technique was successfully applied to the surface treatment of various commercial polymers (polyolefins, polyethers, polyacetylenes, polyimides, etc.) [1,2,3,4]. A majority of the experiments were carried out by interaction with gaseous fluorine (gas-phase fluorination), which are limited by low fluorine concentration, temperature and duration of the treatment due to the constrained heat dissipation and possible ignition of polymeric material. Recently, a liquid-phase fluorination variation of the technique was suggested to overcome the aforementioned restrictions. Here, the almost inert liquid phase (a perfluorinated organic liquid) withdraws the reaction heat and should potentially allow the reaction to be performed under mild conditions. This method has been tested previously on conventional polymers (polyphenyleneoxide [5], substituted celluloses [6], polystyrene [7] and polynorbornene [8]). It was demonstrated that the temperature and fluorine concentration in the fluorinated mixture with helium did not significantly affect the consumption of fluorine, and resulted in surface layers with low fluorine content. This might be partially associated with low mobility of the fluorine in the treated polymeric materials, since the polymers belong to a group of materials with average and low permeability (oxygen permeability coefficient varies in the range of 0.6–24 Barrer [8,9,10,11]). The depth of fluorination has been shown to strongly depend on the diffusion coefficient of fluorine in the polymer precursor [3,12]. Thus, it would be desirable to test polymeric materials with higher permeability.

**Figure 1:**
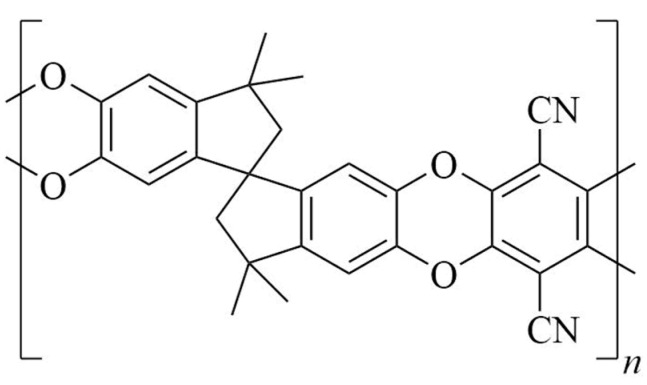
Chemical structure of PIM-1.

Recently, polybenzodioxane PIM-1 (Figure 1), a well-known highly permeable membrane polymer, has been subjected to gas-phase fluorination, and significant improvement in He-CH_4_, He-N_2_ and He-CO_2_ separation performances was demonstrated [13]. This effect can be ascribed to the combination of two causes: (i) improved solubility selectivity and (ii) increased diffusion selectivity. PIM-1 has been also utilized as a polymeric membrane material for nanofiltration [14,15] and pervaporation [16,17] processes. The intrinsic hydrophobicity of PIM-1 membranes promotes the removal of volatile organic compounds from aqueous solutions [18], while its surface hydrophilization improves its anti-fouling property toward colloids, microorganisms and charged inorganic particles [19]. The hydrophobic–hydrophilic balance also determines membrane chemical resistance in liquid media [20]. Meanwhile, direct fluorination is able to adjust the energetic properties of the membrane surface and the hydrophobicity of polymeric materials [21], depending on fluorination conditions. Therefore, the development of approaches for controlled fluorination of membrane materials is an interesting problem, and one of the ways to solve it may be liquid-phase fluorination. It is necessary to understand the effects of liquid-phase fluorination conditions on the degree of surface fluorination, on changes in the surface properties of the material, as well as on the depth of fluorine penetration, to control the fluorination process and to obtain a material with certain characteristics.

In the current investigation, we treat PIM-1 films with elemental fluorine dissolved in liquid perfluorodecalin and perform their characterization by scanning and energy-dispersive X-ray spectroscopy (SEM and XEDS), X-ray photoelectron spectroscopy (XPS), IR spectroscopy and contact angle assessment technique.

## 2. Materials and Methods

### 2.1. Synthesis of PIM-1

A 5.5′,6,6′-tetra-hydroxy-3,3,3′,3′-tetramethyl-1,1′-bispiroindane, tetrafluoroterephthalo-nitrile, carbonate of potassium with the solvents dimethylsufoxide and toluene were loaded into a three-necked flask equipped with a high-speed stirrer and a tube for supplying dry argon. The reaction mixture was stirred for 1–2 min at a speed of 5000 rpm in an argon current, then transferred to a preheated to 60 °C silicone bath and maintained at this temperature and mixing speed (1000 rpm), periodically (hourly) accelerating to 5000–10,000 rpm for 1–2 min. After heating the reaction mixture for 8 h, the stirrer was stopped, the resulting precipitate was filtered out, and then was successively washed with hot 50% ethanol and hot water to remove the potassium fluoride that formed during the polycondensation. The yield of PIM-1 obtained in the form of a fine yellow powder after vacuum drying was not less 90%. The weight averaged molecular mass of the PIM-1 samples determined via GPC (Agilent 1100 setup (Agilent Technologies, Inc., Santa Clara, CA, USA) with UV-VIS detector (wavelength 430 nm), two columns Ultrastyragel Linear, chloroform as eluent, the eluent flux of 1 mL/min and column temperature at 25 °C) corresponded to 75 kDa relatively to polystyrene standards. The polydispersity index of the samples was ~2.2. The detailed investigation of the PIM-1 synthesis was published elsewhere [22,23,24].

### 2.2. Preparation of PIM-1 Samples for Fluorination

The PIM-1 film samples were cast from 3 w/w% solution of PIM-1 (in distilled chloroform). The solution was pipetted into a molding ring with a cellophane bottom. After evaporation of the solvent, the film was carefully removed from the ring and dried under vacuum (~1 mbar) to a constant weight. All samples were placed in plastic bags and sealed to avoid water uptake and prevent rapid aging. The thickness of the films varied in the range of 70–100 μm.

### 2.3. Direct Liquid-Phase Fluorination of PIM-1 Samples

Surface fluorination of PIM-1 was carried out according to the following procedure. A gaseous mixture of fluorine (10 vol.%) and nitrogen (90 vol.%) was bubbled into a 1.5 L chamber made of fluoroplast (hereinafter the reactor) with 350 mL of perfluorodecalin (PFD) at room temperature (22 ± 2 °C) for 60 min to saturate the liquid phase with fluorine and simultaneously to remove dissolved air oxygen. The flow rate was set using a flow meter Bronkhorst F-201EV-AAD-33-K (Bronkhorst, Ruurlo, Netherlands) at 0.5 mL·s^−1^. A magnetic stirrer with 50–60 rpm allowed a mixing of the liquid phase. After the specified time (60 min), the fluorination mixture supply was closed. Then, a sample of PIM-1 was placed into a holder (the diameter of the films was 47 mm) and installed in the reactor with PFD in such a way that surface liquid-phase fluorination took place on one side. One turned on the supply of the fluorination mixture (with flow rate of 0.5 mL·s^−1^) to the reactor and simultaneously started mixing with the magnetic stirrer (50–60 rpm). The temperature of the experiment corresponded to room temperature. After 15 min of processing, the fluorination mixture supply was closed and the magnetic stirrer was turned off. The sample of PIM-1 was taken out of the reactor and dried with a filter paper. The procedure of PIM-1 fluorination was repeated for another three initial PIM-1 samples, but fluorination was performed for 30, 45 and 60 min, respectively. The experiment was carried out twice for each fluorination time.

A film of polybenzodioxane PIM-1 for comparison (control sample) was prepared in the reactor without supply of the fluorination mixture. The PFD was stirred for 30 min using the magnetic stirrer. Then, the surface layer of the PIM-1 film was saturated with PFD. After 30 min, the membrane holder was removed from the reactor and the PIM-1 film was taken out. The residual PFD on the surface of the PIM-1 film was removed by placing the film between two discs of the filter paper.

### 2.4. X-ray Photoelectron Spectroscopy (XPS)

The chemical composition of the fluorinated samples of PIM-1 were tested by X-ray photoelectron spectrometer FLEXPS (Specs, Berlin, Germany). The setup was equipped with a hemispheric electron analyser Phoibos 150 (Specs, Berlin, Germany) and an electron detector with multichannel plate (150 channels) and delay line 1-DLD. Excitation of spectra were carried out by irradiation of Al and Mg Kα of X-ray tube with double anode XR-50 (Specs, Berlin, Germany). The transmission energy of the electron analyzer was 50 eV for a record of overall spectra and 20 eV for that of particular lines. Vacuum in the system was maintained at 10^−10^ mbar. A slow electron (1–3 eV) gun was utilized to offset the charging of samples. The calculation of bond energies was reduced to a C1s line with 284.8 eV of carbon atoms forming covalent bonds C-C or C-H for PIM-1 films according to Gao et al. [25]. Assignment of binding energies for other element lines (O1s, F1s, N1s, Na1s, Cl2p, Si2p) was also proved by means of Scienta ESCA300 Database [26]. The relative atomic concentrations of elements were calculated on the basis of peak areas taking into account cross-sections of electron photoionization, free path length of electrons and electron transmission function. The size of the analyzed surface of the sample was about 2 mm. The depth of the analyses determined by an exponential attenuation of electron energy when exiting the sample and corresponding to 95% of collecting electrons were ~1.1, 0.9 and 0.8 nm for lines of C1s, O1s and F1s respectively. One decomposed the spectra into symmetrical components simulating the convolution of Lorentz and Gaussian lines for detailed consideration. The background of inelastic electron scattering was subtracted using the Shirley method. Deconvolution of the spectra was carried out by CasaXPS v. 2.3.25 software. For the layer-by-layer analysis of PIM-1 films, one applied a RIE 12/38 ion etching gun with an Ar+ ion beam (an energy of 1 keV) to scan a 5 × 5 mm^2^ surface. The ion current was 29 µA. Ion etching was carried out for each sample for 60, 180, 300, 600 and 900 s. After each etching, the XPS spectra were recorded, respectively.

### 2.5. Scanning and Energy-Dispersive X-ray Spectroscopy (SEM and XEDS)

Cross-sections of the untreated and treated PIM-1 films were prepared. The samples were collected in a single package with uniform orientation of the fluorinated sides. The cross-sections surfaces were coated with platinum to ensure electron sink during SEM and XEDS analysis. The electron microscopy and elementary analysis of the sample package was conducted using a Quanta 200 3D scanning electron microscope (FEI, Hillsboro, OR, USA) with a Pegasus materials characterization system (by EDAX, Warrendale, PA, USA). SEM and EDS analysis was performed on the atoms F, O and C. An averaged length of step for XEDS scanning corresponds to 0.3 μm.

**Figure 2:**
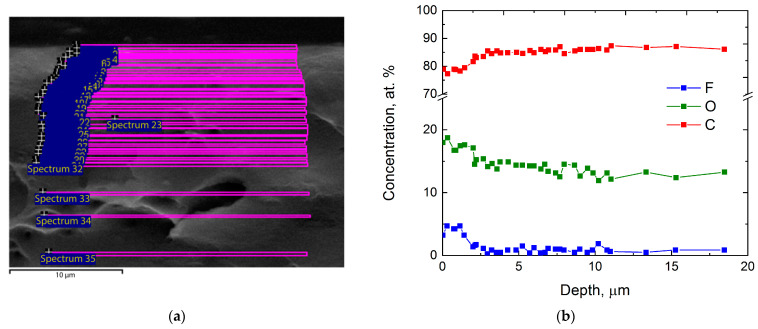
A typical set of results for a single film sample: (**a**) a scanning scheme of cross-section of the sample on a SEM-image, (**b**) graphical representation of distribution of element concentration on the depth.

SEM images of cross-sections of all samples (untreated and treated PIM-1 films) were obtained (Figure 2a). Scanning lines (Figure 2a) were transformed into dependencies of the distribution of element concentration according to their depth (Figure 2b). Figure 2 represents a typical set of results for a single sample.

### 2.6. Attenuated Total Reflectance Infra-Red Spectroscopy (ATR-IR)

ATR-IR spectra of PIM-1 films before and after fluorination were recorded in the mode of attenuated total reflectance (ATR) on IFS 66 v/s IR Fourier spectrometer with an ATR instrument (Ge and ZnSe crystals) in the region of 4000–600 cm^−1^, with a resolution of 2 cm^−1^ and averaging over 50 scans. Use of the Ge crystal allows for scanning of the spectra to a depth of 0.66 μm from the film surface, while the ZnSe crystal allows for a depth of 2.0 μm.

### 2.7. Contact Angle Measurements and Surface Energy Calculation

The advancing contact angles (θ) of two test liquids (H_2_O, CH_2_I_2_) were measured at 20 ± 1 °C using a horizontal microscope equipped with a goniometric attachment. The dispersion (γ^d^_SV_) and polar (γ^p^_SV_) components of the surface free energy (γ_SV_) values of the films were determined by the two-fluid method within the framework of the Owens–Wendt–Kaelble approximation [27,28]. The average values for not less than 6 drops of probe liquids were used for calculation of the contact angles. The measurement accuracy was 1° for contact angles and 1 mJ∙m^−2^ for surface and interfacial energies of the polymer films.

## 3. Results and Discussion

### 3.1. ATR-IR of the Fluorinated PIM-1 Samples

**Figure 3:**
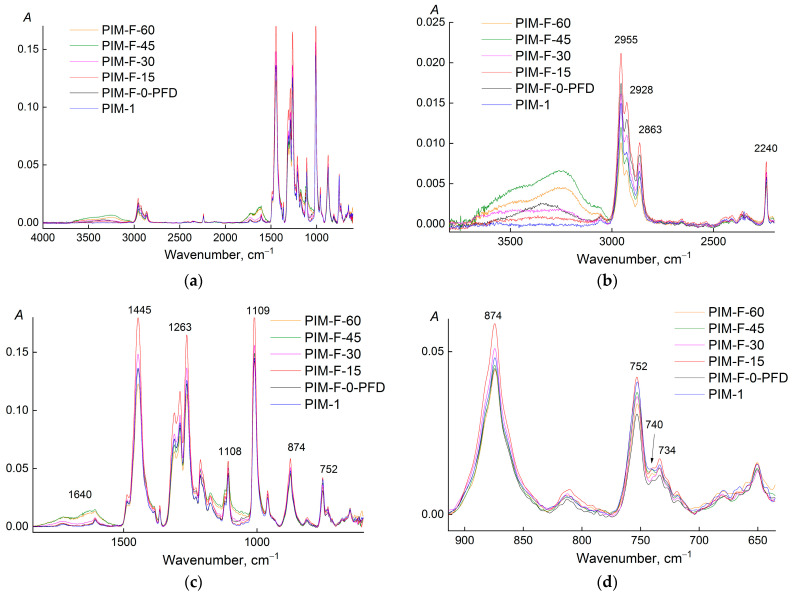
ATR-IR spectra (ZnSe crystal) of virgin PIM-1 film, PIM-1 film soaked in PFD (PIM-F-0-PFD), and PIM-1 films treated by fluorination mixture [F_2_ (10 vol.%) + N_2_] in perfluorodecalin for 15 (PIM-F-15), 30 (PIM-F-30), 45 (PIM-F-45) and 60 min (PIM-F-60) at (22 ± 2) °C for different ranges of wavenumbers. (**a**): full-scale spectra; (**b**): zoomed in 2000–4000 cm^−1^ region of the spectra; (**c**): zoomed in 500–2000 cm^−1^ region of the spectra; (**d**): zoomed in 650–900 cm^−1^ region of the spectra.

The ATR-IR spectra (ZnSe crystal, irradiation penetration depth of 2 μm) of the PIM-1 films before and after fluorination and after exposure to PFD are shown in Figure 3. The exposure of PIM-1 film to PDF has no significant effect on the chemical structure of the surface layer of the film. The intensity of the absorption bands in the region of the wavenumbers of stretching and bending vibrations of water (3400–3200 cm^−1^, 1640 cm^−1^) increases with the fluorination time (Figure 3b,c). In addition, an increase of the baseline was observed in the spectra in the region of 1700–1600 and 1400–1000 cm^−1^ (Figure 3c), where the absorption bands of C=O and C–F bonds are located. Hence, the fluorination of the PIM-1 chemical structure is accompanied by its oxidation.

**Figure 4:**
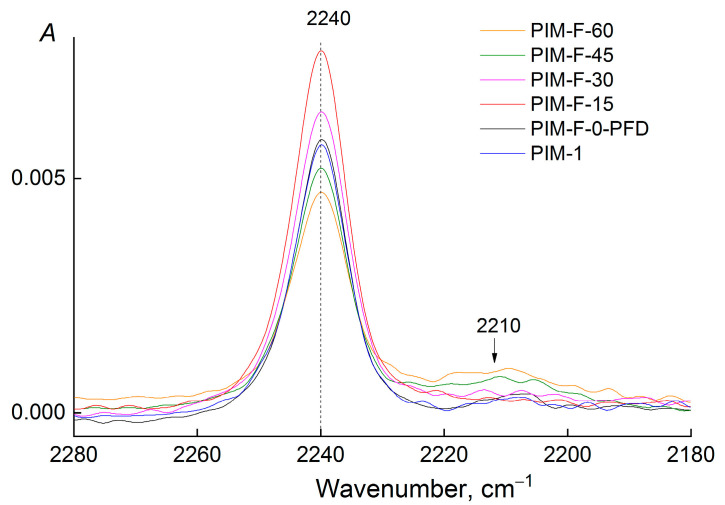
The absorption region of C≡N bonds in the ATR-IR spectra of the films before and after fluorination.

It is impossible to quantify the content of CF-groups in the fluorinated PIM-1 films using ATR-IR spectra. A small concentration of them and the absence of individual C-F absorption bands in the spectra allow us only to estimate the influence of these groups on the neighboring functional groups in the fluorinated chemical structure of PIM-1, and only on the depth of irradiation penetration through the material of the ATR-crystal (for ZnSe it is 2 μm). To estimate the change in the content of functional groups in the surface layer of the PIM-1 film, it is necessary to find an absorption band that can be considered as an internal standard. The absorption band of the stretching vibrations of CN-group (2240 cm^−1^) does not change the position of the maximum and retains the shape at all durations of fluorination. This indicates that the polarization of the CN-bond does not change (Figure 4). Only at 60 min of the fluorination, a low–intensity band (2210 cm^−1^) appears in the long-wavelength region, which is usually explained by a change in CN-bond order [29,30]. However, the intensity of this band (2210 cm^−1^) is low, and the nitrile group does not change sensitively and can be used as an internal standard. Thus, the optical density of all the absorption bands used in the analysis was normalized to the optical density of the absorption band at 2240 cm^−1^.

It should be noted that the absorption band at 874 cm^−1^ (bending vibrations of the unsubstituted H atom in the aromatic ring, Figure 3d) does not change the position of the maximum and shape (there is no splitting). This means that fluorination of protons in the aromatic ring does not occur.

**Figure 5:**
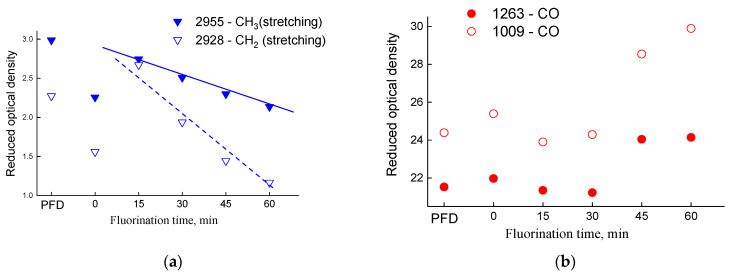

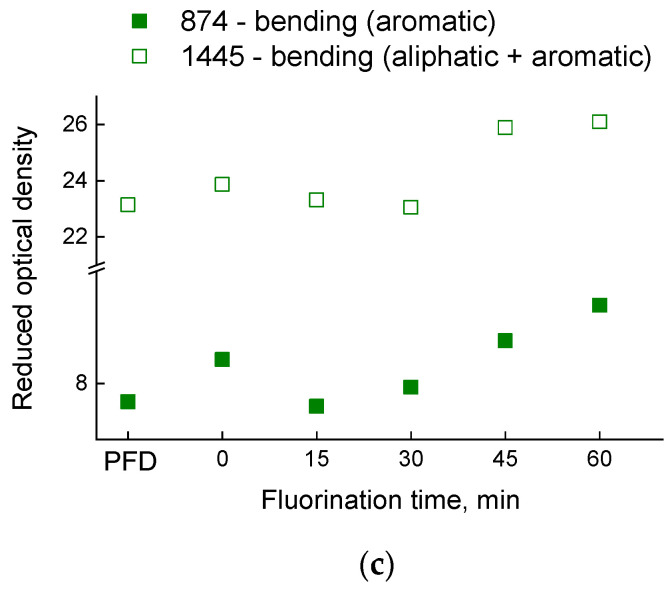
Changes in the optical density of some PIM-1 absorption bands from fluorination time for (**a**) aliphatic stretching vibrations (CH_3_ and CH_2_), (**b**) carbon-oxygen bonds and (**c**) bending vibrations of aromatic (C=C) and aliphatic (CH) groups.

Table 1 shows the values of the reduced optical density of the absorption bands normalized by the intensity of the absorption band of nitrile groups (*A_n_*, where *n* is the wavenumber at the maximum of the absorption band). The change in the optical density of some absorption bands is clearly shown in Figure 5.

For the PIM-1 sample fluorinated for 15 min, the intensity of the absorption bands of the stretching vibrations of the CH bonds increases sharply (2955 and 2928 cm^−1^), then the optical density of these absorption bands decreases linearly (Figure 5a) and is minimal at 60 min of fluorination. At the same time, the rate of consumption of methylene groups is higher than that of methyl groups. Therefore, fluorination (and/or oxidation) of methylene groups takes place first.

The optical density of the absorption band of bending vibrations (1445 cm^−1^ in Figure 5c) decreases during the first 15 min of fluorination, then increases. The intensity of this absorption band is contributed to both by bending vibrations of aliphatic CH groups and plane vibrations of the C=C bonds of aromatic structures. During fluorination (and/or oxidation) of methylene and methyl groups, the contribution of stretching vibrations from C=C in the aromatic ring increases, which leads to an increase of the intensity of this absorption band (Figure 5c). At the same time, the presence of F (or O) in substituents will change the polarizability of bonds nearby functional groups, which will affect the change in their intensity. One observes such an increase in Figure 5c for the absorption band at 874 cm^−1^ (stretching vibrations of the unsubstituted H atom in the aromatic ring). This fact confirms the assumption that the protons of the aromatic ring are resistant to fluorination: otherwise, with a decrease of the number of protons in the aromatic ring, the intensity of this absorption band should have decreased.

### 3.2. XPS Analysis of the Fluorinated PIM-1 Samples

**Figure 6:**
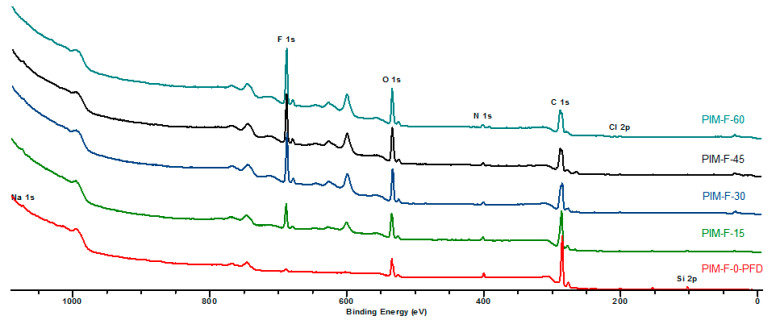
The overall XPS spectra of the surfaces of the control sample (PIM-F-0-PFD) and PIM-1 samples fluorinated for 15 (PIM-F-15), 30 (PIM-F-30), 45 (PIM-F-45) and 60 min (PIM-F-60).

Figure 6 shows the overall spectra of the fluorinated PIM-1 films. C, O, F, N, Cl, Na, Si atoms were detected on the surface of the samples. Chlorine may be present in the spectra as a trace of residual solvent after the evaporation of the polymer solution whereas sodium and silicone can be considered contaminants appearing during the sample preparation for the XPS analysis.

**Figure 7:**
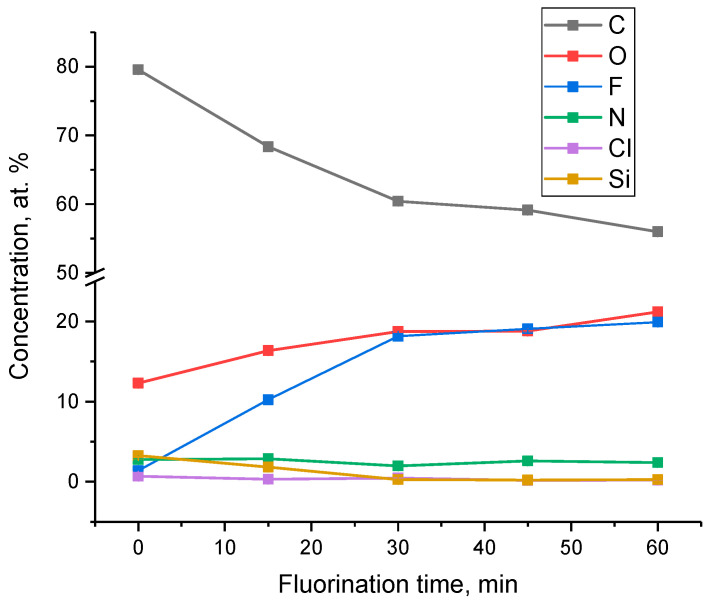
Change in the concentration of elements for surface of PIM-1 samples determined by XPS depending on the fluorination time.

The atomic concentrations of the elements obtained from the results of XPS measurements are shown in Table 2. Surprisingly, the fluorine is detected in the control sample (Table 2, Figure 7), which indicates the penetration of perfluorodecalin into the film during nitrogen bubbling without fluorination. The concentration of fluorine and oxygen increases with the fluorination time and reaches a plateau. This trend clearly shows accumulation of fluorine- and oxygen-containing functional groups in the surface layer of the PIM-1 films, which stabilizes in ~1.0 nm depth layer after 30 min of fluorination. It should be pointed out that the changes of intensities of the stretching vibrations in ATR-IR spectra of aliphatic methyl-, methylene- and C-O groups in the ~2.0 μm depth layer gradually decreased. Therefore, this indicates that no layers with uniform concentration of elements are formed, and the fluorinated layer has variable element content along the depth.

The concentration of carbon decreases and also reaches a plateau while the concentration of nitrogen practically does not change (Figure 7). It should be pointed out that the decline and stabilization of carbon atom content in the surface layer is mostly associated with the corresponding behavior of fluorine and oxygen contents. Chlorine and silicon atoms, which concentration gradually decreases with increase of the fluorination time, are also present in the film.

**Figure 8:**
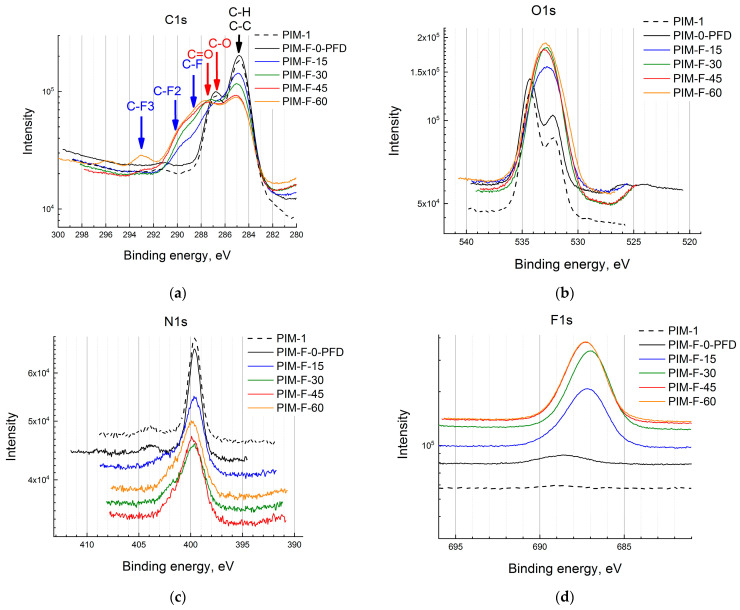
XPS spectra of C1s (**a**), O1s (**b**), N1s (**c**) and F1s (**d**) bands in the PIM-1 sample for control sample (PIM-F-0-PFD) and PIM-1 films fluorinated for 15 (PIM-1-F-15), 30 (PIM-1-F-30), 45 (PIM-1-F-45) and 60 min (PIM-1-F-60).

The change in the chemical state of carbon atoms after fluorination is shown in Figure 8a. For the sample PIM-F-0-PFD, there are two main lines at 284.8 eV (C-C/C-H) and at 286.7 eV from ether C-O bonds. C-N line of cyano-groups at 285.7 eV has low intensity and does not detected in the C1s spectrum (Figure 8a). The line at 287.3 eV corresponds to C=O bond. The intensity of the line increases and its position shifts to 287.7 eV with increasing of the treatment time. This behavior of the band can be associated with the formation of the C-F bond that appears at 288.7 eV. The intensity of this line increases, and it shifts to 289.5 eV with the increase of the fluorination time. At the longest fluorination times, additional components at 290.5 eV and ~293 eV appear, which can be associated with the formation of C-F_2_ and C-F_3_ bonds, respectively. The lines at ~289 eV can be also associated with the formation of a carboxyl group.

Two main components are observed at 534.3 eV and 532.1 eV in the spectra of the O1s level (Figure 8b). Oxygen atoms from the C-O-C bond, as well as adsorbed water molecules, contribute to the component with a higher binding energy. A line with a lower binding energy is associated with the C=O functional group, which is also observed in the C1s spectrum. A low-intensity line at 532.7 eV, detected only when the spectra are decomposed into components, can be associated with surface C-OH group. A new line appears at 533.7 eV (in addition to the line at 532.1 eV) in the samples after fluorination. It can be assumed that along with the contribution from the C-O-C group, two oxygen atoms from the carboxyl group, the formation of which is noted in the C1s spectra, contribute to these lines.

The N1s spectrum of the control sample is represented by a single line at 399.6 eV (Figure 8c), which is typical for the nitrile group [26]. The low intensity peak at the 404 eV region for the fluorinated samples results in a widening of the peak and is associated with oxidized nitrogen atoms.

A single line at 689 eV having negligible intensity is detected in the F1s spectrum of the virgin PIM-1 sample (Figure 8d). This line can be attributed to the presence of small amounts of fluorine atoms that were not involved in polycondensation and correspond to the terminal groups of PIM-1 macromolecular chains. After liquid-phase fluorination of PIM-1 samples, a new intense component appears at 687.2 eV corresponding to fluorine atoms in the C-F groups. Its position does not depend on the fluorination time. The intensity of the line gradually increases with the fluorination time and almost does not change for the sample fluorinated for 60 min.

### 3.3. SEM and XEDS for the Fluorinated PIM-1 Samples

**Figure 9:**
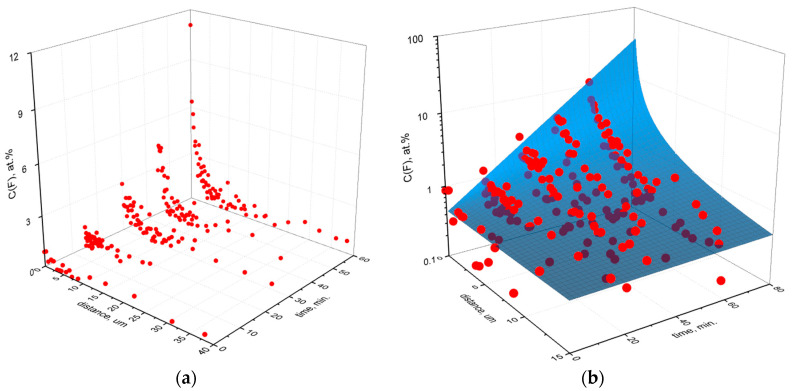
Experimental data of the XEDS study (**a**) and surface (**b**) of the distribution of the fluorine content C(F) over the depth (distance from the surface) of the PIM-1 films depending on the fluorination time.

The fluorination time (*t*_F_) dependence of the change in the content of fluorine atoms C(F) over the thickness L of the PIM-1 films is presented in Figure 9a. It was pointed out previously that in the case of low and middle permeable polymers (cellulose acetate [9], polynorbornene [8], polystyrene [11,31], etc.), a sharp boundary between the fluorinated layer and bulk polymer was detected by IR-spectroscopy [32], SEM and XEDS [8,9] techniques. The fluorine concentration profiles (Figure 9a) show that C(F) is maximal on the surface of the films and gradually decreases as the depth increases. This means that no sharp boundary exists in the case of fluorinated PIM-1 samples. However, a fast decrease of fluorine concentration finishes within several microns depending on the fluorination time. An approximate depth of fluorination for the sample fluorinated for 60 min is 6–7 µm. The more precise estimations are difficult due to the high scatter of the fluorine concentrations (Figure 9a).

It is also worth mentioning that for different treatment durations, the values of fluorine concentration at the surface (Figure 9a) are somewhat lower than those obtained by XPS technique (Table 2). This observation is reasonable because, as was previously mentioned, XPS technique has the depth of analysis within 1 nm and monitors the very surface layer (see Section 2.4), while XEDS has a scanning spot size of about 1 μm [9].

All data on the diagram in Figure 9a are fitted by a functional dependence using least-square method:

(1) $$C\left(F\right)=A\cdot \exp\left(B\cdot t_{F}\right)\cdot L^{-C\cdot t_{F}}$$

where, C(F) is the atomic concentration of fluorine (at.%), t_F_ is the fluorination time (min), A, B and C are constants equal to 0.4586, 0.0347 and 0.0125, respectively. The dependence of fluorine content on film depth and fluorination time is graphically described by a surface presented in Figure 9b.

At zero fluorination time, the content of fluorine equals the value of the coefficient A, which characterizes the value of the “initial” fluorine content in the film, independent of external influence, or reflects the value of the “background” signal of the detector. In any of these interpretations, the value of the coefficient A can be considered a conditional “zero” level at the maximum depth of penetration of fluorine at different times of fluorination. Taking into consideration that the control sample of PIM-1 was soaked in perfluorodecalin, the “zero” concentration A may reflect the fluorine concentration due to the presence of the perfluorinated solvent (liquid phase for the fluorination). Formulas to determine the maximum depth *L*_0_, at which the time derivative of the function (Equation (1)) is equal to zero, are the following:

(2) $$\frac{dC\left(F\right)}{dt}=\frac{A\cdot B\cdot \exp\left(B\cdot t_{F}\right)-A\cdot C\cdot \exp\left(B\cdot t_{F}\right)\cdot \mathrm{lnL}}{L^{C\cdot t_{F}}}=0,$$

(3) $$L_{0}=\exp\left(\frac{B}{C}\right).$$

The maximum depth of penetration *L*_0_ of fluorine into the PIM-1 film at any fluorination time does not exceed 16 μm. This value is rather theoretical, serves as an estimate of the depth of liquid phase (perfluorodecalin) penetration into the PIM-1 film samples, and should be taken into consideration for interpretation of other physicochemical parameters of the surface fluorinated PIM-1 films.

### 3.4. Surface Properties of Fluorinated PIM-1 Films

The results of contact angle measurements are provided in Table 3. The initial surface of PIM-1 is wetted by water to a limited extent. After contact with perfluorodecalin, the surface becomes slightly more hydrophobic, which may be due to the presence of a residual PFD in the polymer film. Nevertheless, soaking in perfluorodecalin does not change the surface free energy of polymer film (PIM-F-0-PFD compared to PIM-1), which, as expected, appeared to be close to the surface energy of polystyrene (γ_SV_ = 44.0 ± 4.5 mJ∙m^−2^) [33]. After 15 min of fluorination, the dispersion component of the surface energy sharply decreases, which is due to the appearance of fluorine-containing groups on the surface [34] and is consistent with the IR data. The surface energy of PIM-F-15 is close to that of tetrafluoroethylene (γ_SV_ = 19.6 mJ∙m^−2^) [28]. In this case, a slight increase of the polar component of the surface energy is observed, which indicates the appearance of polar groups on the surface [34] as a result of oxidative processes. An increase of the fluorination time leads to hydrophilization of the surface and a gradual increase of the polar component of the surface energy. It indicates the predominant contribution of oxidative processes rather than fluorination to the change in the surface properties, as the latter should have provided an increase of the hydrophobicity of the surface and a decrease of its polarity [35].

## 4. Conclusions

The direct liquid-phase fluorination for 15, 30, 45 and 60 min was performed on dense polybenzodioxane PIM-1 films. The analysis of the surface fluorinated samples showed that the fluorine is introduced to the polymeric films via absorption of perfluorodecalin and chemical reaction of fluorine with functional groups of macromolecular chains. The fluorination of the surface layer is accompanied by oxidation of the chemical structure. According to XPS data, the fluorine and oxygen contents in the near-surface layer (1 nm depth) were gradually increased up to ~20 at.% with the fluorination time. Hydrogen-substitution fluorination was conducted predominantly in methylene- and methyl-groups of PIM-1 polymer structure, while aromatic hydrogens, aromatic structure and cyano-groups were rather stable under liquid-phase fluorination conditions. According to SEM-XEDS analysis of the fluorinated samples, fluorination progressed to the different depths from the surface of the films. The estimated depth of fluorination corresponds to 6–7 microns for the films fluorinated for 60 min. The model taking into account the fluorination duration and distance from the surface of polymeric film was suggested. It might be useful for the further investigations of fluorination of polymeric materials kinetics. It was found that the fluorination time is a parameter which allows for the varying of the surface energy of PIM-1 film from a low value, corresponding to Teflon, up to a high value, corresponding to a hydrophilic high-energy surface. Surface oxidation and hydrophilization begin to prevail over the hydrophobic effect of fluorine atoms (which reduce the specific surface free energy) presence at the surface at fluorination times greater than 15 min. Therefore, the liquid-phase fluorination technique can be recommended for tailoring hydrophobic-hydrophilic surface properties of PIM-1 materials used in nanofiltration, pervaporation and other applications.

## Figures and Tables

**Table 1:** Reduced optical density (*A_n_*) for PIM-1 films depending on the fluorination time; *n* is the wavenumber at the maximum of the absorption band.

Fluorination Time, min	*A_n_*							
	*А*_2955_	*А*_2928_	*А*_1445_	*А*_1309_	*А*_1263_	*А*_874_	*А*_752_	*А*_734_
0 (virgin)	2.26	1.56	23.87	13.17	21.98	8.43	7.12	2.67
0 (PFD)	2.99	2.27	23.15	12.83	21.53	7.68	5.29	2.08
15	2.74	2.67	23.32	12.68	21.35	7.60	5.47	2.22
30	2.51	1.94	23.06	12.36	21.23	7.93	5.61	2.24
45	2.30	1.44	25.90	13.48	24.04	8.76	7.18	2.73
60	2.14	1.17	26.10	13.69	24.14	9.39	7.23	3.03

**Table 2:** Atomic concentrations (%) of C, O, F, and N in PIM-1 samples depending on fluorination time according to XPS data.

Fluorination Time, min	C	O	N	F
0 (virgin)	82.3	13.5	3.9	0.2
0 (PFD)	82.8	12.8	2.9	1.5
15	69.9	16.8	3.0	10.4
30	60.9	18.9	2.0	18.2
45	59.3	18.9	2.6	19.2
60	56.3	21.3	2.4	20.0

**Table 3:** Changes in the characteristics of the surface of PIM-1 films depending on the conditions of fluorination.

Sample	*θ*(H_2_O), deg	*θ*(CH_2_I_2_), deg	*γ*^d^_SV_ mJ∙m^−2^	*γ*^p^_SV_, mJ∙m^−2^	*γ*_SV_, mJ∙m^−2^
PIM-1	89	25	46	1	47
PIM-F-0-PFD	93	26	47	*close to zero*	47
PIM-F-15	91	67	21	4	25
PIM-F-30	70	38	34	9	43
PIM-F-45	36	- *	- *	- *	- *
PIM-F-60	22	- *	- *	- *	- *

* The energy characteristics of PIM-F-45 and PIM-F-60 were not calculated, since the contact angles of water on the surface of these samples appeared to be small. In this case, the Owens–Wendt–Kaelble approach is not correct to apply [36].

## Data Availability

Not applicable.
